# Dogs with a vocabulary of object labels retain them for at least 2 years

**DOI:** 10.1098/rsbl.2024.0208

**Published:** 2024-09-04

**Authors:** Shany Dror, Ádám Miklósi, Claudia Fugazza

**Affiliations:** ^1^ Department of Ethology, Eötvös Loránd University, Pázmány P. s 1c, 6th Floor, Budapest 1117, Hungary; ^2^ Messerli Research Institute, University of Veterinary Medicine, Vienna, Austria; ^3^ ELTE NAP, Comparative Ethology Research Group, Budapest, Hungary; ^4^ MTA-ELTE, Comparative Ethology Research Group, Budapest, Hungary

**Keywords:** object label learning, word memory, gifted word learner dogs, long-term memory

## Abstract

Long-term memory of words has a crucial role in the developing abilities of young children to acquire language. In dogs, the ability to learn object labels is present in only a small group of uniquely gifted word learner (GWL) dogs. As they are very rare, little is known about the mechanisms through which they acquire such large vocabularies. In the current study, we tested the ability of five GWL dogs to retrieve 12 labelled objects 2 years after the object-label mapping acquisition. The dogs proved to remember the labels of between three and nine objects. The results shed light on the process by which GWL dogs acquire an exceptionally large vocabulary of object names. As memory plays a crucial role in language development, these dogs provide a unique opportunity to study label retention in a non-linguistic species.

## 1. Introduction

The ability to remember newly learned words has a central role in the process of infants’ vocabulary development. Word learning is the formation of a link between a word form and its meaning [1]. The perceptual trace of this new mapping is then consolidated to maintain it over a longer period of time. Recall occurs when the memory trace is reactivated [2]. While multiple instances of word learning should lead to the development of a vocabulary [1], this process would not be possible without consolidating the mappings of the newly learned words. Therefore, although many studies focus on speed of word acquisition, it is of equal importance to understand memory retention capacities [2]. In young children, different developmental trajectories appear for encoding, consolidating and recalling of words, and these processes may involve different mechanisms or networks in the brain [3]. The rapid increase in infants word acquisition at the age of 18 months likely reflects a shift in their ability to consolidate and retrieve previously encoded words [2].

Studies on the ability of infants and young children to remember words referring to object labels often examine their ability to recall the labels after a brief exposure, with delays varying from 24 h to several days or one month. Before the onset of word production, 7.5-month-old infants can recognize the phonological patterns of a word 24 h after they have been familiarized with it [4]. Between the ages of 1 and 2 years, infants can retain novel object labels for at least 24 h (for review refer [2]). Carey & Bartlett [5] demonstrated that after a single exposure, 3-year-olds retained the memory of a word that was used to refer to an object for 7–10 days. Other studies have shown that at the age of 3 and 4 years, children can remember a new object label for at least one month, even after a brief exposure [6]. As long-term memory has a large role in language development, it would be of great interest to understand the memory capacities of non-human species for labels and their mappings, as this may help shed light on which aspects of language are domain general and which are specific to humans’ linguistic abilities [7].

Among the various potential animal models that could be used for studying language-related cognitive skills, dogs are a particularly valid model because they evolved and develop in the human society [8]. Furthermore, family dogs in Western societies often live in an environment similar to that of a developing child and are exposed to human language. Multiple studies have examined the ability of dogs to solve short-term memory tasks, with delays ranging from several seconds to 1 h (e.g. [9–12]). However, studies examining long-term memory are less common. One study examining deferred imitation found that dogs can reproduce a presented action after a delay of 24 h [13]. A study examining the ability of dogs to remember a learned action has shown that after being exposed to a long training procedure, consisting of 18 sessions, dogs retained the memory of the learned action for at least one month [14]. Dogs that were trained over a period of several months on a visual discrimination task on a touchscreen were able to remember the task six months after they were last exposed to the stimuli [15]. Dogs trained for scent detection tasks are able to remember a target scent for which they received multiple exposures, for 1 year after the initial training [16]. A study examining kin recognition found that mothers exhibited a preference for a cloth containing scent from their adult offspring, 21–22 months after separation [17]. Thus, after multiple exposures, dogs can form long-term memories of olfactory or visual stimuli, as well as action commands, but very little is known about their ability to remember object labels.

Several studies found that the majority of dogs do not show behavioural evidence of learning object labels [18–22], and studies conducted with dogs that already possess a vocabulary of object labels normally include a small sample of 1–3 dogs (e.g. [23–26] but refer [27] for an attempt to reach a larger sample). Thus, the studies conducted so far suggest that the ability to retrieve objects based on their labels is present in only a small group of gifted word learner (GWL) dogs [22,27]. Consequently, very little is known about the mechanisms through which dogs form object label mappings and how they retain these in their memory. GWL dogs are known for their ability to rapidly achieve large vocabularies of object labels [27,28], raising the hypothesis that in a manner functionally similar to children, they may be able to retain the object-label mappings in their long-term memory. However, while children may experience extended periods without encountering a previously learned word, GWL dogs typically play with their owners with the labelled objects on a daily basis [27]. In this case, the frequent reintroduction of the objects and their labels may enable the formation of the large vocabulary and reduce the need for forming long-term retention of object labels. In a previous study [28], we demonstrated that GWL dogs are able to retain object-label mappings for a period of at least two months.

In the current study, we asked whether the same dogs will still remember those object labels after 2 years have elapsed since they last heard the labels and had access to the objects. As the ability to retain the memory of stimuli for a period of over 1 year has already been demonstrated in dogs in other domains (e.g. [16,17]), we hypothesized that the GWL dogs consolidate object verbal labels and retain those for considerably long periods of time.

## 2. Methods

### (a) Subjects

Five GWL dogs (two females, average age 6.6 ± 2 years (± s.d.), all border collies) participated in the study. All of the dogs have participated in Dror *et al*. [28].

### (b) Objects

The dog toys for this test were the same toys used in [28] and a detailed description of the methods can be found in that article and in the electronic supplementary material, Methods. During December 2020, the dogs were taught the names of 12 toys in 1 week. During this week, the amount of time the owners spent interacting with their dogs with the objects varied between owners as it depended on the owners’ availability. Three owners (of the dogs Max, Rico and Squall) reported spending no more than 30 min per day on the task, one owner (of the dog Whisky) reported spending up to 1.5 h per day and one owner (of Gaia) spent up to 5.5 h. At the end of that week, the dogs were tested on their knowledge. After one month, we tested the dogs’ retention of the labels of six of the toys randomly selected among the 12. The other six toys were used in an object label retention test that occurred two months after the objects were stored. After each test, the owners were asked to store the toys out of the dogs reach for 2 years. Between February and June 2023, we tested the dogs’ retention of the object labels (see testing procedures below). Out of the five dogs, two owners misplaced some of the toys and therefore, one dog was tested on only 11 toys (Squall) and one on only five (Rico). For the 2-year retention test presented in the current study, the toys were randomly divided into two sets of six, each of which was tested on a different occasion. To make sure that during the test the dogs did not get overexcited due to the presence of the reintroduced toys, 1 day before the test the owners were instructed to leave the toys on the floor and allow the dogs to inspect those for 1 h. During this time, the owners were instructed not to engage with the dogs. On the day of the test, 1 h before it, the dogs again received 30 min of access to the toys.

### (c) Experimental set-up

The experimental set-up was the same as that used for experiments 3 and 4 in [28]. Tests were conducted online. The owners placed the toys in a room and sat in an adjacent room, out of view from the toys. Two video recording devices, one in each room, were connected to an online platform, so that the experimenters could observe the dogs’ behaviour in real time.

### (d) Testing procedure

One set including six toys randomly chosen among the 12 (test toys) was placed on the floor together with eight toys from the dog’s collection. The toys from the dog’s collection were used to reduce the dogs’ excitement level, increase the possible choice set and encourage searching behaviour. The toys from the dog’s collection were not used for data analysis. The owners asked the dog to retrieve each toy by saying the objects label (e.g. ‘Can you get <object label>?’). The six test toys were asked one after the other in a semi-random order. Each toy was requested two times (overall 12 trials for all dogs except Rico, as he had only five toys, and therefore had only 10 trials). Whenever there were only three toys left on the floor, the owner placed all the toys back. This way the number of test toys from which the dog could choose always varied between six and four (disregarding the presence of the eight toys from the dog’s collection). In cases where the dogs made a correct choice, the owner praised the dog, played with him/her with the toy (without saying the toy’s label) or gave the dog a treat. In cases where the dogs made incorrect choices, the test was briefly interrupted as the owner went to the room with the toys and removed the toy that was not retrieved. For Gaia, Max and Squall, one week after the test, another identical test was carried out with the second set of test toys. As Rico only had five toys, he was tested only once. Due to owner availability, for Whisky, both tests were conducted on the same day (one in the morning and one in the evening). A video presenting an example of the testing procedure is available at this link.

### (e) Data collection and analysis

The dogs correct or incorrect choices were coded during the test. Data analysis was performed using the R statistical environment v. 4.2.2 [29] in RStudio [30]. When calculating chance level, we conservatively ignored the presence of the eight toys from the dog’s collection and only considered the six test toys. Chance level was first calculated for each dog individually, by calculating the average number of test toys that were placed on the floor in each trial (refer to [31] for a similar method of calculating chance level). The group average chance level was then calculated from the individual chance levels. To determine whether the dogs as a group, performed above chance, we used a Wilcoxon signed-rank test, comparing the dogs’ average performance to the average chance level. Binomial tests were used to determine the individual probability of each dog retrieving the test toys, using the dog’s individual chance level.

We used Wilcoxon signed-rank tests to compare the dog’s performance in the current study to their performance in the one- and two-month memory tests in [28]. Since, in Dror *et al*. [28], the dogs were requested to retrieve each toy three times, for each toy tested in 2021, we considered only the first two trials in which it was requested. An additional analysis of the first and second trials in which the toys were requested is available in the electronic supplementary material.

## 3. Results

### (a) Group performance

The average group performance was 44% (±14) correct choices. The group average chance level was 20.4%. As a group, the dogs performed significantly above chance (Wilcoxon signed-rank test; group average versus group chance level, *W* = 15, *p* = 0.029).

### (b) Individual performance

Four out of the five dogs performed significantly above chance (binomial test, all significant *p* ≤ 0.016, see table 1). These dogs successfully retrieved between three and nine of the reintroduced toys at least once.

**Table 1.** The dog’s individual performance during the 2-year memory retention test (binomial tests, the dogs’ average chance level was calculated based on the average number of toys from which the dog could choose).

**d**og	toys tested	no. toys retrieved once	**no.** toys retrieved twice	**s**um of toys retrieved	**p**roportion of correct trials	**a**verage chance level	***p-*value**
Gaia	12	5	4	9	13/24 (54%)	19.8%	<0.001
Max	12	6	2	8	10/24 (41%)	20.2%	0.013
Whisky	12	4	3	7	10/24 (41%)	19.7%	0.016
Squall	11	3	1	4	5/22 (23%)	20.7%	0.441
Rico	5	—	3	3	6/10 (60%)	21.7%	0.009

### (c) Comparison between the 2-year memory test and the monthly memory tests conducted in Dror *et al*., (2021)

**Figure 1.**
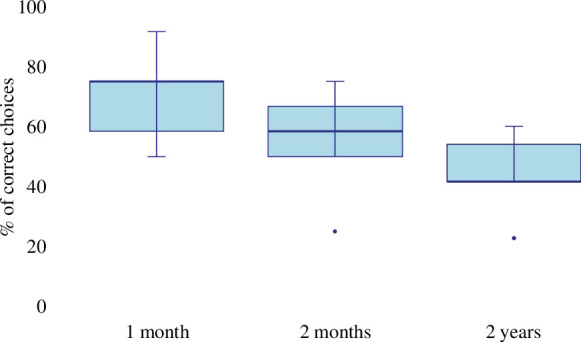
Percentage of the dog’s correct choices in the one- and two-month memory tests published in [28], and the 2-year memory test.

In the one- and two-month memory tests conducted in [28], the average performance for these five dogs was 70% (±16) and 55% (±19), respectively (figure 1). In the 2-year memory test, the dogs’ average performance was 44% (±14). The dogs’ performance in the 2-year memory test did not differ from their performance in the one-month memory test but presented a trend towards significance (Wilcoxon signed-rank, *V* = 15, *p* = 0.0625). Their performance in the 2-year memory test did not differ from their performance in the two-month memory test (Wilcoxon signed-rank, *V* = 14, *p* = 0.125).

## 4. Discussion

Our findings show that, after initial consolidation, most of the GWL dogs tested in this study maintained the object-label mappings over a 2-year time period, without any further rehearsal. Four out of the five GWL dogs recalled the labels of between three and nine toys. The findings presented here reveal that, in addition to the rapid learning rates described in previous studies [21,27,28], GWL dogs also form a long-term memory of the object-label mappings after short exposures that enable them to accumulate large vocabularies. When comparing the dogs’ group performance in the present 2-year memory test, with that of the one- and two-month memory tests, it appears that there was no significant reduction in their recall of the labelled objects. However, the fact that there was a trend towards significance in the comparison between the 2-year and one-month memory tests, raises the possibility that larger sample sizes may reveal a reduction in the dogs’ recall over such a long timeframe.

Wojcik [32] showed that in 3- to 6-year-old children, the number of novel labels presented during learning affected later retrieval. In the current study, the GWL dogs were asked to recall a large number of words that were all introduced within a short time period. Despite these conditions being especially challenging, the GWL dogs did demonstrate an ability to recall some of the object labels. Some of the objects were retrieved on both test trials, which may suggest that the dogs had an especially stronger memory for these object-label mappings. The positive feedback that the dogs received after a correct choice may have also facilitated the recall procedure in subsequent trials.

It is likely that reducing the initial number of labels to which the dogs were exposed, or increasing the duration of the exposure to the object label pairings, would have led to an even better performance in the recall test. Indeed, the dog whose owner reported longer daily interactions (Gaia) demonstrated a slightly higher performance.

In humans, retrieval practice of a previously learned novel word can improve long-term memory [33]. As the GWL dogs were tested on their ability to recall the labelled objects one and two months after the initial exposure, this may have facilitated the recall of these object labels 2 years after the last test.

Little is known about the long-term memory capacities of the domestic dog. Our findings expand our knowledge of this topic by showing that some individual dogs can maintain object-label mappings years after they have first been exposed to them. However, from the fact that this capacity is within the species’ cognitive abilities, we cannot infer that it is a common characteristic. As the majority of family dogs do not show behavioural evidence of learning object labels [18,19], the findings presented here cannot be generalized to other dog populations or other cognitive domains. Furthermore, as this study was conducted with only a small sample of five GWL dogs, with some variations in the testing and training protocol between individuals, we could not examine potential factors that may influence the dogs’ ability to learn and recall object labels.

Our findings suggest that the ability to retain object label mappings in long-term memory is a domain general ability which, at least at a functional level, is not uniquely human. In humans, high performance in verbal memory tasks has been associated with high-performance in other memory tasks, such as visuospatial memory and autobiographical memory [34], and other cognitive domains, such as executive functioning [35]. GWL dogs provide a unique opportunity to examine whether such correlations may also exist in a species that does not possess language. Any similarities or differences found between the manner by which GWL dogs and humans form long-term memories of labels may help us better understand how the different cognitive abilities evolved and interlocked in the human brain to form language as we know it.

## Data Availability

Supplementary data and methods are available on Dryad [36]. Supplementary material is available online [37].
